# Exchange biased delta-E effect enables the detection of low frequency pT magnetic fields with simultaneous localization

**DOI:** 10.1038/s41598-021-84415-2

**Published:** 2021-03-05

**Authors:** B. Spetzler, C. Bald, P. Durdaut, J. Reermann, C. Kirchhof, A. Teplyuk, D. Meyners, E. Quandt, M. Höft, G. Schmidt, F. Faupel

**Affiliations:** 1 Institute of Materials Science, Faculty of Engineering, Kiel University, Kaiserstraße 2, 24143 Kiel, Germany; 2 Institute of Electrical Engineering and Information Technology, Faculty of Engineering, Kiel University, Kaiserstraße 2, 24143 Kiel, Germany

**Keywords:** Sensors and biosensors, Magnetic properties and materials

## Abstract

Delta-E effect sensors are based on magnetoelectric resonators that detune in a magnetic field due to the delta-E effect of the magnetostrictive material. In recent years, such sensors have shown the potential to detect small amplitude and low-frequency magnetic fields. Yet, they all require external magnetic bias fields for optimal operation, which is highly detrimental to their application. Here, we solve this problem by combining the delta-E effect with exchange biased multilayers and operate the resonator in a low-loss torsion mode. It is comprehensively analyzed experimentally and theoretically using various kinds of models. Due to the exchange bias, no external magnetic bias fields are required, but still low detection limits down to ${{\text{350 pT}} \mathord{\left/ {\vphantom {{\text{350 pT}} {\sqrt {{\text{Hz}}} }}} \right. \kern-\nulldelimiterspace} {\sqrt {{\text{Hz}}} }}$ at 25 Hz are achieved. The potential of this concept is demonstrated with a new operating scheme that permits simultaneous measurement and localization, which is especially desirable for typical biomedical inverse solution problems. The sensor is localized with a minimum spatial resolution of 1 cm while measuring a low-frequency magnetic test signal that can be well reconstructed. Overall, we demonstrate that this class of magnetic field sensors is a significant step towards first biomedical applications and compact large number sensor arrays.

## Introduction

The detection of low-frequency magnetic fields and the localization of its source is of interest for many biological and biomedical applications^1–3^. Because these fields result in very small signal amplitudes in the pT regime and below, SQUID magnetometers^4^ are traditionally used. These sensors are extensive and expensive in operation, as they base on superconducting devices that must be cooled and magnetically well-shielded during the measurements. Significant progress has been made with optically pumped magnetometers^5,6^. They sense magnetic fields by the change of transmission of a laser beam through a gas cell upon application of a magnetic field. With this concept detection limits in the order of a few ${{{\text{fT}}} \mathord{\left/ {\vphantom {{{\text{fT}}} {\sqrt {{\text{Hz}}} }}} \right. \kern-\nulldelimiterspace} {\sqrt {{\text{Hz}}} }}$ were achieved in a frequency range from about 1–100 Hz^7^. Although their handling is improved compared to SQUID magnetometers, they still require a magnetically shielded environment and external temperature control. For both types of sensors, the limited integrability and the comparatively large size limit the number of sensors that can be used in typical biomedical array applications. Such applications are mainly inverse solution problems, such as MEG source localization^8^ or source imaging of the heart^9^. For a correct solution the precise knowledge of the sensor’s position and orientation is essential^10^ as well as a large number of measurements.

In recent years, strain-mediated magnetoelectric composite magnetic field sensors have been investigated for similar purposes^11–13^. They consist of mechanically coupled magnetostrictive and piezoelectric materials and can be processed on a large scale by MEMS technology with dimensions of a few millimeters^14^ down to a few micrometers^15,16^. Such small devices are of special interest for array applications due to the potentially improved spatial resolution and reduced equipment costs.

Utilizing the magnetoelectric effect^12^, limits of detection < 1pT/${\sqrt {{\text{Hz}}}}$ can be reached^17^ with cm sized sensors, but only in mechanical resonance. Hence, the low detection limits are restricted to rather high-frequency magnetic fields within a narrow bandwidth around the device’s resonance frequency. Such high frequencies and small bandwidths do not match the requirements of many biomedical applications.

One way to overcome the limitations is the utilization of modulation techniques used e.g. in the delta-E effect read-out scheme. The delta-E effect of the magnetic material^18–21^, is the change of the Young’s modulus upon application of a magnetic field or a mechanical stress. Using the sensor concept with plate and cantilever magnetoelectric resonators, detection limits in the sub-nT regime have been achieved in frequency bandwidths up to 100 Hz^16,22–25^. Such low detection limits are achieved by applying a magnetic bias field with external coils to operate the sensor at its optimum signal-to-noise ratio, close to the maximum curvature of the magnetostriction curve. This comes at the expense of integrability and size of the sensor system with direct consequences for the application.

In general, coils require additional electronics and are difficult to integrate, which diminishes the advantage of using integrable technology for the magnetoelectric resonators. In previously presented delta-E effect sensors^16,22–25^, the coils increase the size of the system to several centimeters, even though the actual resonators have only dimensions in the hundreds of µm to mm range. As a result, the minimum distance of sensor and source increases, which can reduce the measured signal amplitude significantly. Moreover, the size and stray fields of the coils can be problematic for building dense, large number sensor arrays that are desirable for many biological and biomedical applications. Consequently, replacing the coils is an important step towards application.

A few devices avoid external magnetic bias fields using magnetic hysteresis^16,22^ at the expense of the detection limit. Magnetic hysteresis is connected with statistical magnetization or domain reorientation processes, which are intrinsically linked to magnetic noise^26^. Consequently, this approach is potentially problematic for the reproducibility, stability, and the noise performance of magnetic field sensors.

A different way of achieving self-biased systems is the implementation of exchange biased multilayers^27,28^. Such multilayers consist of a sequence of antiferromagnetic and ferromagnetic layers. The magnetization of these layers is coupled at their interface by a unidirectional exchange interaction that defines a preferable direction of the magnetization in the ferromagnetic layers^29^. Overall, the effect on the ferromagnetic layers is similar to an external magnetic field.

Here we report on mm-sized delta-E effect sensors based on exchange biased multilayers and analyze their potential for the detection of small amplitude and low-frequency magnetic fields. A domain model^30^ is extended to describe the magnetoelastic properties of the multilayer. Various other models are used to analyze measurements of the electromechanical and the sensing characteristics. Further, we demonstrate an operation technique that combines the advantages of direct magnetoelectric and delta-E operation. It permits the detection of low-frequency magnetic fields using the delta-E effect in a higher resonance mode (RM), while simultaneously using the direct detection of the first mode to localize the sensor. The simultaneous measurement of location and signal is expected to be especially advantageous if the source can move, which is typically the case for source localization or imaging on patients. As a proof of concept, we use this technique to measure the magnetic field of a low frequency magnetic test signal with the delta-E effect, while localizing the sensor via the direct detection scheme.

## Results

### Exchange biased MEMS sensor

**Figure 1:**
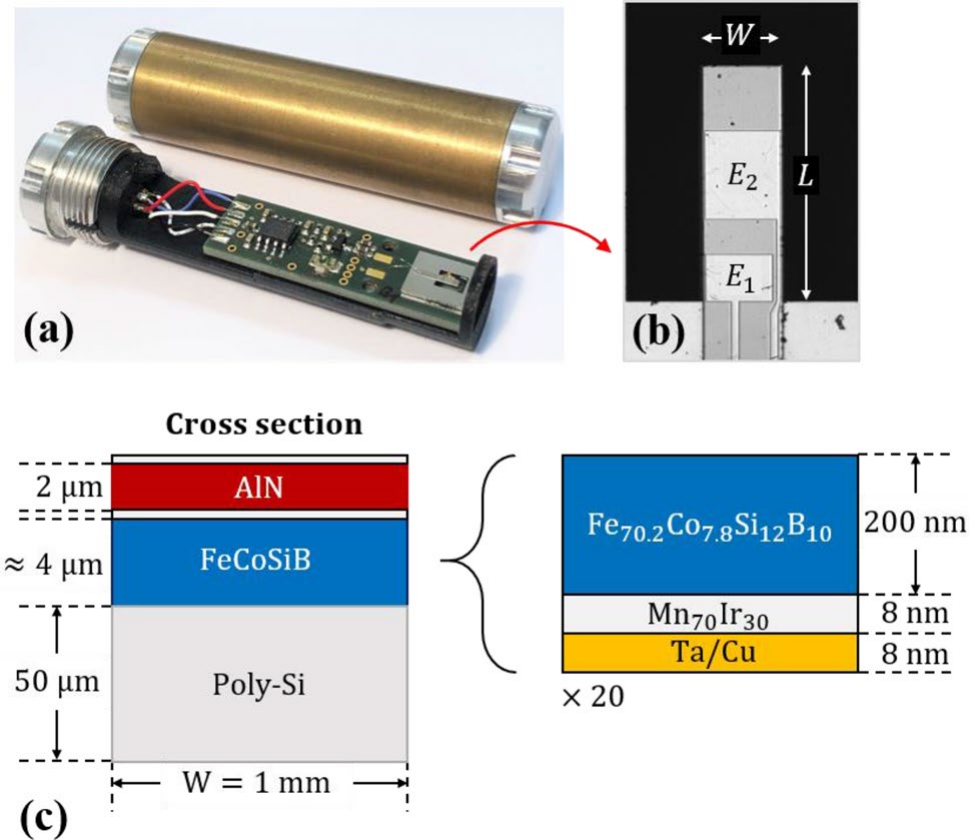
Exchange biased delta-E effect sensor. (**a**) Exchange biased magnetoelectric composite cantilever sensors presented in this study with (top) and without (bottom) encapsulation. The devices consist of a MEMS chip with a magnetoelectric resonator on a printed circuit board (PCB) with a JFET charge amplifier^32^. The PCB is mounted on a 3D-printed holder (black) with a ring at its end for mechanical protection of the cantilever. A brass encapsulation (brass thickness: 2.1 mm) is used for electrical shielding. (**b**) Microscopy image of a cantilever (*W* = 1 mm, *L* = 3 mm) with top electrodes *E*_1_ and *E*_2_. (**c**) Schematic cross section of the cantilever with thicknesses of the functional layers and the structure of the exchange biased magnetic multilayer. Details are given in the “Materials and methods” section.

The magnetoelectric composite sensors presented in this study are made by MEMS technology on a polysilicon cantilever surrounded by a silicon frame (Fig. 1a). The $W = {\text{1 mm}}$ wide, $L = 3{\text{ mm}}$ long, and 50 µm thick cantilevers are covered by a magnetic multilayer and a piezoelectric AlN layer (Fig. 1c). The magnetic layer is made of a sequence of $20 \times ({\text{Ta / Cu / Mn}}_{70} {\text{Ir}}_{30} /{\text{Fe}}_{70.2} {\text{Co}}_{7.8} {\text{Si}}_{12} {\text{B}}_{10} )$ with a total thickness of about 4 µm. The multilayer is tempered under application of a strong magnetic field applied at an angle of 55° relative to the long cantilever axis. This process induces a magnetic easy axis and sets the exchange bias field. The 2 µm thick AlN layer^31^ on top of the magnetic film is used for excitation and read-out simultaneously. It is sandwiched by two Ta-Pt electrodes. An adapted electrode design^23^ permits the efficient excitation of the 1st and 2nd bending mode. In this study both electrodes $E_{1}$ and $E_{2}$ (Fig. 1b) are used. Process details are described in the “Materials and methods” section. As shown in Fig. 1a, the MEMS chip with the magnetoelectric cantilever is placed on a printed circuit board (PCB) and connected to a low noise JFET charge amplifier. Its equivalent circuit is given in Ref.^32^. The PCB is mounted on a 3D-printed holder and encapsulated in a brass cylinder (brass thickness: 2.1 mm) for electrical shielding and mechanical protection.

### Magnetic properties

For the analysis of the magnetic properties, a magnetoelastic model is built. Simple single-domain models are accompanied by strong magnetic hysteresis for magnetic fields that are not applied along the magnetic hard axis^33^. In soft-magnetic FeCoSiB thin-films, domain wall motion is expected to dominate the magnetization reversal process^26^. This causes a significantly smaller coercive field than predicted by single-domain Stoner-Wohlfarth models, especially for magnetic fields applied close to the magnetic easy axis. Consequently, domain wall motion must be considered to describe the magnetization and the delta-E effect. The few numerical domain models that exist^30,34^ include neither exchange bias fields, nor arbitrary magnetic field directions and are therefore extended by the respective energy contributions (“Materials and methods” section).

**Figure 2:**
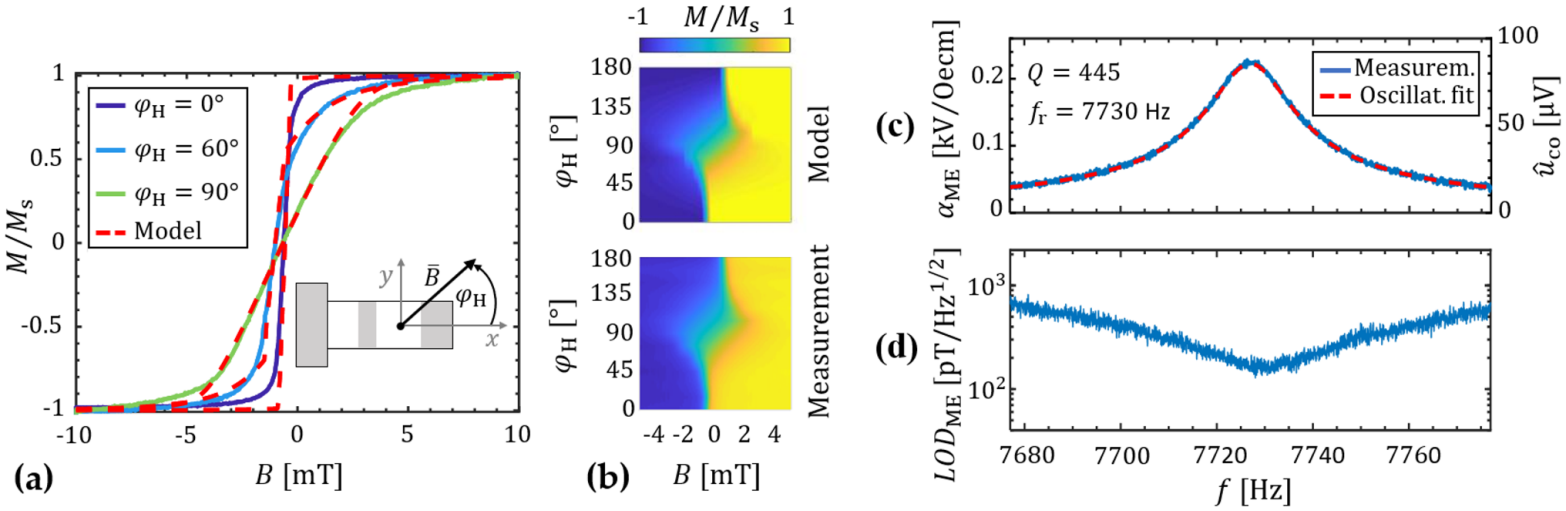
Magnetic and magnetoelectric properties. (**a**) Magnetization loops measured with a BH-loop tracer in three different directions are compared with the simulations: along the long axis $(\varphi_{{\text{H}}} = 0^\circ )$, the short axis $(\varphi_{{\text{H}}} = 90^\circ )$, and an example orientation in between $(\varphi_{{\text{H}}} = 60^\circ )$. For the exchange bias field a magnitude $B_{{{\text{ex}}}} \approx {0}{\text{.8 mT}}$ and orientation of $(\varphi_{{{\text{ex}}}} = 46^\circ )$ is obtained. (**b**) Comparison of measured and modeled magnetization curves in the range of $\varphi_{{\text{H}}} = 0^\circ - 180^\circ$. (**c**) Magnetoelectric coefficient $\alpha_{{{\text{ME}}}}$ of the first bending mode (RM1), calculated via Eq. (1) from the measurements. The ac magnetic flux density is applied along the x-axis (**a**) with an amplitude of $\hat{B}_{{{\text{ac}}}} = {\text{100 nT}}$. From a harmonic oscillator fit a quality factor of $Q_{1} \approx 445$ and a resonance frequency of about $f_{{\text{r,1}}} = {\text{7728 Hz}}$ are calculated. (**d**) Detection limit measured around the first bending mode. A minimum value of about $LOD_{{{\text{ME}}}} = {\text{150 pT/}}\sqrt {{\text{Hz}}}$ is obtained at $f_{{\text{r,1}}}$ with encapsulation, and an estimated value of approximately *LOD*_ME_ = 30 pT/Hz without.

In Fig. 2a measured and modeled magnetization curves are compared for a magnetic flux density $\overline{B}$ applied along the long axis $(\varphi_{{\text{H}}} = 0^\circ )$, the short axis $(\varphi_{{\text{H}}} = 90^\circ )$, and for an example angle in between $(\varphi_{{\text{H}}} = 60^\circ )$. The model matches the measurements well. Only small deviations occur around the transition from a dominant wall motion to a dominant moment rotation, which results in a larger curvature of the modeled magnetization curves. In a real multi-domain sample, the spatially distributed magnetic domains and effective anisotropies are expected to smooth out this transition. Overall, the model does reproduce the measured magnetization behavior correctly, even over the full range of $\varphi_{{\text{H}}}$ (Fig. 2b). An effective easy axis orientation of $\theta = 1.5^\circ$ relative to the x-axis is found from the fit, with an effective uniaxial anisotropy energy density of *K*_u_ = 1.8 kJ/m^3^. The exchange bias field is found to be oriented $\varphi_{{{\text{ex}}}} = 46^\circ$ with a magnitude $B_{{{\text{ex}}}} \approx {0}{\text{.8 mT}}$. This is identical to the orientation and magnitude found experimentally from the x-axis offset of the two measured hysteresis curves along $\varphi_{{\text{H}}} = 0^\circ$ and $\varphi_{{\text{H}}} = 90^\circ$.

### Direct operation

For the sensor localization, the direct detection mode will be used. It utilizes the direct magnetoelectric effect in the first resonance mode (RM1), the first bending mode. The magnetoelectric effect is often characterized by the magnetoelectric coefficient

1 $$\alpha_{{{\text{ME}}}} = \frac{{\hat{u}_{{\text{p}}} }}{{t_{{\text{p}}} \hat{B}_{{{\text{ac}}}} }}{\text{, with }}\hat{u}_{{\text{p}}} = \hat{u}_{{{\text{co}}}} \frac{{C_{{\text{f}}} }}{{C_{{{\text{ME}}}} }}$$

which can be calculated from the voltage amplitude $\hat{u}_{{\text{p}}}$ across the piezoelectric layer, the thickness $t_{{\text{p}}}$ of the piezoelectric layer and the ac amplitude $\hat{B}_{{{\text{ac}}}}$ of the field to be measured, applied along the x-axis (Fig. 2). The voltage amplitude $\hat{u}_{{\text{p}}}$ across the piezoelectric layer relates to the output voltage amplitude $\hat{u}_{{{\text{co}}}}$ of the charge amplifier via the feedback capacitance $C_{{\text{f}}} = {\text{33 pF}}$ of the charge amplifier and the capacitance $C_{{{\text{ME}}}}$ of the magnetoelectric sensor, which is determined in the following section as $C_{{{\text{ME}}}} = {\text{63 pF}}$. All amplitudes in this paper are tagged with a hat in contrast to root mean square values.

For the characterization of the first bending mode (RM1) in the direct detection mode, a sinusoidal magnetic field was applied along the x-axis with an amplitude $\hat{B}_{{{\text{ac}}}} = {\text{100 nT}}$ and frequencies $f_{{{\text{ac}}}}$ around the expected first bending resonance frequency. The magnetoelectric coefficient $\alpha_{{{\text{ME}}}}$ is calculated with Eq. (1) and a harmonic oscillator fit is used to characterize the resonance mode (“Materials and methods” section). The results are plotted in Fig. 2c. In resonance of the first bending mode we obtain $\alpha_{{{\text{ME}}}} = {0}{\text{.22 kV/(Oe}} \cdot {\text{cm)}} = 220{\text{ MV/(T}} \cdot {\text{m)}}$ and a voltage sensitivity at the output of the charge amplifier of $S_{{{\text{ME}}}} = {\text{856 V/T}}$. A quality factor of $Q_{1} \approx 445$ and a resonance frequency of $f_{{\text{r,1}}} = {\text{7728 Hz}}$ are obtained from the harmonic oscillator fit. This yields a -3 dB signal bandwidth of $bw_{1} \approx {8}{\text{.6 Hz}}$ via $bw_{1} = f_{{\text{r}}} /(2Q)$^35^. With an additional noise measurement, we achieve a limit of detection of about $LOD_{{{\text{ME}}}} = {\text{150 pT/}}\sqrt {{\text{Hz}}}$ (Fig. 2d) for the direct detection in resonance. The voltage sensitivity and the limit of detection are in the same range but slightly improved compared to recent values of smaller, externally biased devices^36^. It is worth noting that the ac magnetic field is damped due to the − 3 dB cut-off frequency of the brass cylinder at 1.5 kHz. Without the cylinder, the ME voltage sensitivity around the resonance frequency increases by a factor of about 5, which improves the limit of detection to an estimated value of approximately *LOD*_ME_ = 30 pT/Hz. Here, a limit of detection in the lower pT-regime is not required, because the direct detection mode is only used for the sensor localization.

### Delta-E operation

For the detection of low-frequency magnetic fields, the sensor is operated in delta-E mode. During the delta-E operation, a sinusoidal voltage is applied to the piezoelectric layer to drive the resonator close to its mechanical resonance frequency. A change in the magnetic field leads to a shift of the resonance frequency due to the delta-E effect. This frequency shift leads to a corresponding change of the sensor’s electrical admittance. Hence, an alternating magnetic field modulates the current through the sensor, which can be measured as a voltage $u_{{{\text{co}}}}$ with a charge amplifier. In general, the modulation occurs in amplitude and in phase, depending on the excitation frequency $f_{{{\text{ex}}}}$. In our case, the operating point is chosen such that the phase modulation can be neglected in good approximation. For small signals, the output voltage at the charge amplifier is then approximately:

2 $$u_{{{\text{co}}}} (t) \approx \left| {Z_{{\text{f}}} (f_{{{\text{ex}}}} )} \right| \cdot \hat{u}_{{{\text{ex}}}} \cdot \left[ {Y_{0} + S_{{{\text{dyn}}}} S_{{{\text{am}}}} B_{{{\text{ac}}}} (t)} \right] \cdot \cos (2\pi f_{{{\text{ex}}}} t).$$

Here $\hat{u}_{{{\text{ex}}}}$ is the amplitude of the excitation voltage and $\left| {Z_{{\text{f}}} (f_{{{\text{ex}}}} )} \right|$ the impedance magnitude of the charge amplifier’s feedback network. In the equation, $Y_{0} : = \left| {Y(f_{{{\text{ex}}}} ,B_{0} )} \right|$ is the magnitude of the electrical admittance at $f_{{{\text{ex}}}}$ and the magnetic dc bias flux density $B_{0}$ optionally applied in x-direction during operation or for characterization. The alternating magnetic flux density $B_{{{\text{ac}}}}$ along the x-axis modulates the amplitude $\hat{u}_{{{\text{co}}}}$ of $u_{{{\text{co}}}}$ via the amplitude sensitivity $S_{{{\text{am}}}}$. The sensor’s bandpass characteristic is included in $S_{{{\text{dyn}}}}$ and can be described by a first-order Bessel filter^37^.We define the amplitude sensitivity in accordance with^38^ as:

3 $$S_{{{\text{am}}}} = S_{{{\text{mag}}}} S_{{{\text{el}}}} : = \left. {\frac{{df_{{\text{r}}} }}{dB}} \right|_{{B = B_{0} }} \left. {\frac{d\left| Y \right|}{{df}}} \right|_{{f = f_{{{\text{ex}}}} ,B = B_{0} }}$$

with the electrical amplitude sensitivity $S_{{{\text{el}}}}$ and the magnitude *B* of the dc flux density along the x-axis. The magnetic sensitivity $S_{{{\text{mag}}}}$ can be obtained from the slope of the resonance frequency $f_{{\text{r}}}$ as a function of the applied magnetic flux density *B* at $B_{0}$. To compare the electric and magnetic sensitivities of different devices, relative sensitivities $S_{{\text{mag,r}}} : = (df_{{\text{r}}} /dB)f_{{\text{r}}}^{ - 1}$ and $S_{{\text{el,r}}} : = (d\left| Y \right|/df)f_{{\text{r}}}$ are defined. To reconstruct the magnetic measurement signal $B_{{{\text{ac}}}} (t)$, the charge amplifier’s output signal $u_{{{\text{co}}}} (t)$ is fed into a quadrature amplitude demodulator. Its output voltage is given by

4 $$u(t) = \frac{1}{2}\left| {Z_{{\text{f}}} (f_{{{\text{ex}}}} )} \right| \cdot \hat{u}_{{{\text{ex}}}} \cdot \left[ {Y_{0} + S_{{{\text{dyn}}}} S_{{{\text{am}}}} B_{{{\text{ac}}}} (t)} \right],$$

yielding a voltage sensitivity of

5 $$S_{{\text{V}}} = \frac{1}{2}\left| {Z_{{\text{f}}} (f_{{{\text{ex}}}} )} \right| \cdot \hat{u}_{{{\text{ex}}}} \cdot S_{{{\text{am}}}} S_{{{\text{dyn}}}}$$

The limit of detection (*LOD*), also reffered to as equivalent magnetic noise floor or detectivity can be estimated by

6 $$LOD(f) = \frac{U(f)}{{S_{{\text{V}}} (f)}},$$

With *U* being the voltage noise spectral density at the demodulator’s output for $B_{{{\text{ac}}}} = 0$. For the delta-E operation we use a higher resonance mode (RM). Compared to the first bending mode (RM1) the higher resonance frequency yields a larger signal bandwidth and previous studies have shown a superior sensitivity and *LOD*^23,38^. To find the optimum operating point parameters for the delta-E operation, the admittance *Y* is measured as a function of the excitation frequency $f_{{{\text{ex}}}}$ over a frequency range in which the second bending mode is expected**.**

**Figure 3:**
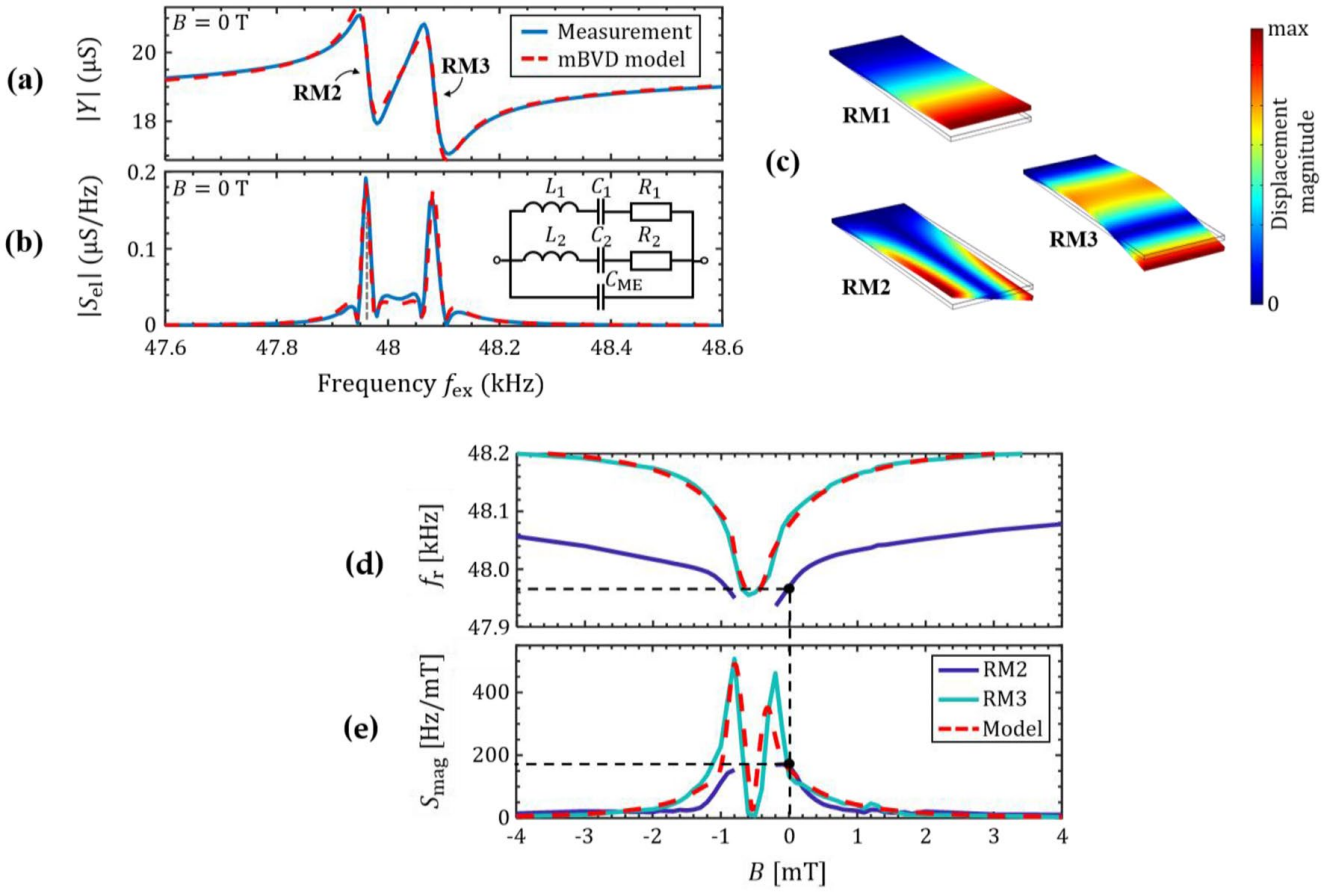
Sensitivity of delta-E operation. (**a**) Measured and modeled electrical admittance of the sensor at *B* = 0 and (**b**) the absolute of its derivative. The modified BVD equivalence circuit used for the fit is shown as inset and the resulting values are given in Table 1. (**c**) Solutions of the finite element study for the first three resonance modes (RM1-RM3) with eigenfrequencies $f_{{\text{r,1}}} < f_{{\text{r,2}}} < f_{{\text{r,3}}}$, (**d**) Simulation and measurements of the resonance frequencies and (**e**) its first derivative the magnetic sensitivity $S_{{{\text{mag}}}}$, as a function of the magnetic bias field applied along the x-axis. The black, dashed lines mark the frequency of operation at zero bias and the corresponding magnetic sensitivity $S_{{{\text{mag}}}}$ and resonance frequency. The measurement was started in the negative magnetic field regime along the cantilever’s long axis.

The measurement is performed with an excitation amplitude of $\hat{u}_{{{\text{ex}}}} = {\text{650 mV}}$ and a magnetic bias field of *B* = 0 mT. As shown in Fig. 3a, the measurement reveals two resonance modes, we refer to as RM2 and RM3, approximately 100 Hz apart from each other. To analyze these modes a modified Butterworth van Dyke (mBVD) model^39^ is fitted to the data. Within this model, each resonance mode is described by an LCR series circuit, both in parallel to each other and to the sensor capacitance $C_{{{\text{ME}}}}$. A sketch of the mBVD model is shown in Fig. 3b together with the magnitude $\left| {S_{{{\text{el}}}} } \right|$ of the electrical sensitivity derived from $\left| Y \right|$. The model fits the measurements well with the parameters given in Table 1. From these parameters, the resonance frequencies $f_{{\text{r}}} = (2\pi \sqrt {LC} )^{ - 1}$ and the quality factors $Q = R^{ - 1} \sqrt {L/C}$ are calculated.

**Table 1:** Results of the mBVD fit. Equivalent circuit parameters, resonance frequencies $f_{{\text{r}}}$ and quality factors *Q* of the two higher resonance modes RM2 and RM3 at zero bias field.

	$f_{{\text{r}}}\text{ }$[kHz]	*Q*	*L* [kH]	*C* [fF]	*R* [${\text{k}}\Omega$]	$C_{{{\text{ME }}}}$[pF]
RM2	47.961	1410	1.4114	7.8	301.68	63
RM3	48.082	1156	0.9937	11	259.62

The resonance frequencies are found to be $f_{{\text{r,2}}} = {47}{\text{.961 kHz}}$ and $f_{{\text{r,3}}} = {48}{\text{.082 kHz}}$. Both modes exhibit high quality factors of $Q_{2} \approx 1400$ and $Q_{3} \approx 1160$, respectively. Especially $Q_{2}$ is higher compared to typical values of $Q \approx 1000$^22,23,25^ for cantilever sensors of a comparable geometry in the first and second bending mode. Among other influence factors, the quality factor depends on the local magnetic properties^22,40^, and significantly on the air damping of the specific resonance mode^41^. Hence, the higher *Q*_2_ of RM2 could indicate the excitation of a different kind of resonance mode.

With a finite element-based eigenfrequency study (“Materials and methods”) the eigenfrequencies of the first three resonance modes (RM1-RM3) of the beam are calculated. Consistent with the measurements, we find the first bending mode at about 7700 Hz and the second bending mode and the first torsional mode both at about 48 kHz. The resulting mode shapes are shown in Fig. 3c. Details on the model and the material parameters used are given in the “Materials and methods” section. As a consequence of the high Q-factors, the − 3 dB bandwidths $bw = f_{{\text{r}}} /(2Q)$^35^ with values of $bw_{2} = {\text{17 Hz}}$ and $bw_{3} = 21{\text{ Hz}}$ are slightly smaller than typical bandwidths of the second bending mode. A maximum electrical sensitivity of $S_{\text{el}} \approx {0.2}{\upmu} \text{S/Hz}(\text{S}_{\text{el,r}} \approx 1~\text{S}/\%)$ is found at an excitation frequency of $f_{{{\text{ex}}}} = 47.96{\text{ kHz}} \approx f_{{\text{r,2}}}$. To analyze the magnetic sensitivities $S_{{{\text{mag}}}}$ of the two resonance modes, the sensor’s admittance is measured for different magnetic bias flux densities *B* applied along the x-axis (Fig. 2), starting close to negative magnetic saturation at B = − 8 mT. The resonance frequencies $f_{{\text{r}}}$ are then extracted with the mBVD model and plotted in Fig. 3d.

With the Euler–Bernoulli eigenfrequency equation (“Materials and methods” section) of the second bending mode we calculate $f_{{\text{r}}}$ using the Young’s modulus *E(B)* predicted by the domain model [“Materials and methods”, Eq. (16)]. Using the same model parameters as in Fig. 2 leads to an excellent match between modeled and measured $f_{{\text{r}}} (B)$ curve of RM3 in Fig. 3d and e. The only parameter altered is the effective anisotropy energy density, which is reduced to *K* = 1160 J/m^3^. The smaller *K*_u_ could be caused by the simplified domain structure of the model, but it is also expected because the 2nd bending mode was found to weight the magnetic properties locally and thereby avoid regions of large effective anisotropy energy densities at the clamping of the cantilever^38^.

Whereas for RM3, $f_{{\text{r}}} (B)$ data are given over the complete range of *B*, no data are shown for RM2 between − 0.9 and − 0.2 mT. In this flux density range, the resonance mode RM2 is no longer excited and hence, not present in the measured admittance characteristic. It is observed in all sensors investigated here and not a unique property of this sample. The magnetic sensitivity $S_{{{\text{mag}}}}$, as defined in Eq. (3) is calculated from the measured and modeled data in Fig. 5a and plotted in Fig. 3e. Whereas the maximum magnetic sensitivity of RM2 is located at zero bias field, for RM3 it is shifted to -0.8 mT. This is a direct consequence of the implemented exchange bias and well reflected by the domain model. At this bias field (− 0.8 mT) the magnetic sensitivity of RM3 is $S_{\text{mag}} \approx 500~\text{Hz}/\text{mT}~(S_{\text{mag,r}} \approx 1\%/\text{mT})$. The magnetic sensitivity $S_{{{\text{mag}}}}$ at *B* = 0 is approximately the same for both resonance modes. Due to the exchange bias it is non-zero and with $S_{{{\text{mag}}}} \approx 175{\text{ Hz/mT (}}S_{{\text{mag,r}}} \approx 0.36{\%\text{/mT)}}$ only a factor of about three times smaller than the maximum value at − 0.8 mT. Using Eq. (3) the amplitude sensitivities of RM2 and RM3 are $S_{{\text{am,2}}} \approx 35 \text{ } \upmu{\text{S/mT}}$ and $S_{{\text{am,3}}} \approx {31.5 \text{ } \upmu} {\text{S/mT}}$ at *B* = 0. These sensitivity values are within the typical range measured at the optimum bias field of non-exchange biased delta-E effect sensors^22,23,25,42^. Consequently, the implemented exchange bias enables high sensitivity delta-E measurements without an externally applied magnetic bias field.

**Figure 4:**
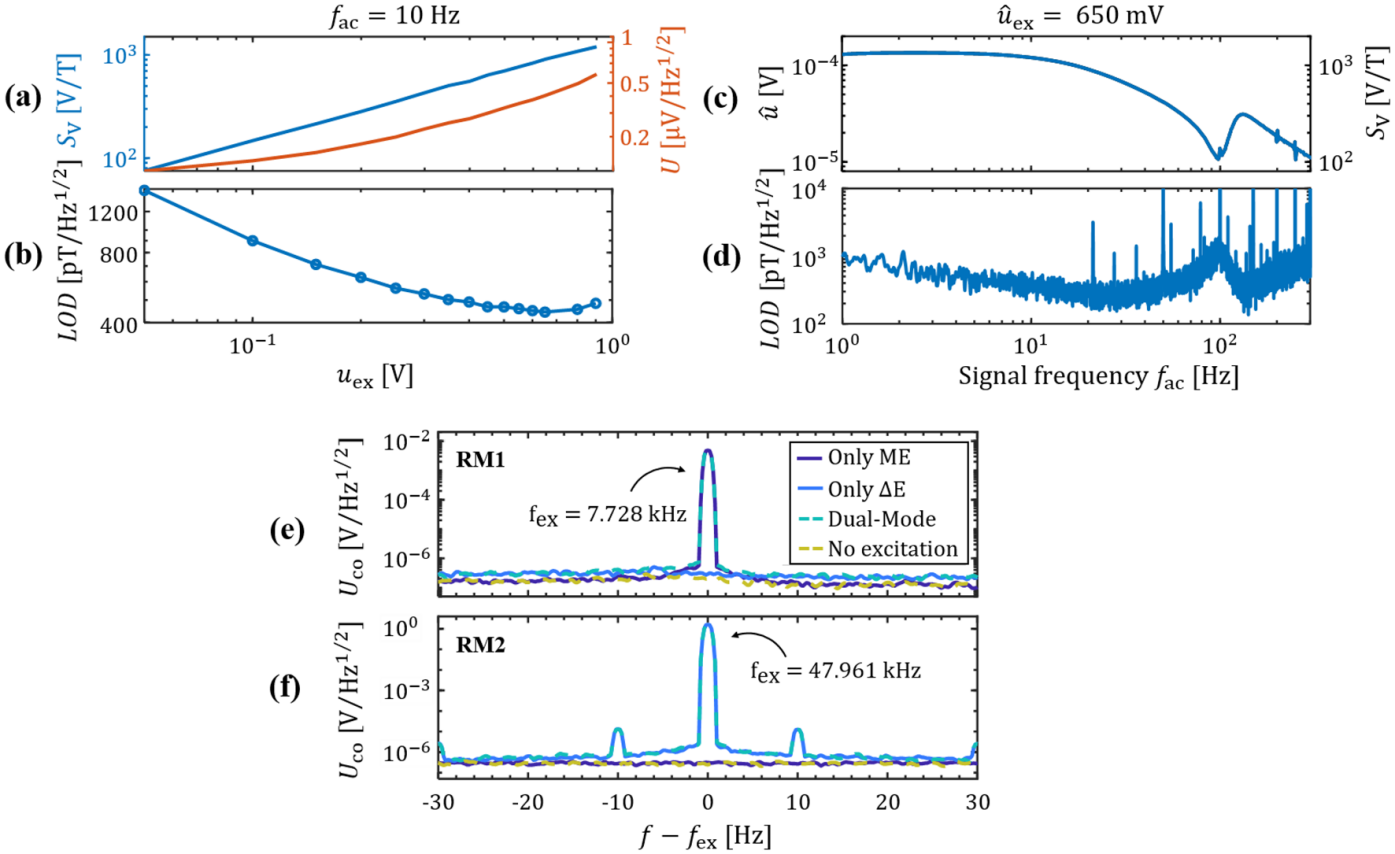
Signal, noise, and LOD. (**a**) Delta-E voltage sensitivity $S_{{\text{V}}}$ and demodulated voltage noise density *U* at *f* = 10 Hz and *B* = 0 as functions of the excitation voltage amplitude $\hat{u}_{{{\text{ex}}}}$, and (**b**) the corresponding limit of detection with a minimum of about $450{\text{ pT/}}\sqrt {{\text{Hz}}}$ found at an optimal excitation amplitude of $\hat{u}_{{{\text{ex}}}} = 650{\text{ mV}}$. (**c**) Signal amplitude and (**d**) LOD, both at the optimal operation conditions of $\hat{u}_{{{\text{ex}}}} = 650{\text{ mV}}$ as functions of the signal frequency, with a minimum of about $350{\text{ pT/}}\sqrt {{\text{Hz}}}$ at 25 Hz**.** The disturbances visible for $f_{{{\text{ac}}}} > 200{\text{ Hz}}$ are caused by insufficient electrical shielding. (**e**) and (**f**) Amplitude density spectra of the sensor for only direct detection (Only ME), only delta-E detection with a 10 Hz test signal (Only $\Delta E$), parallel operation (Dual-Mode) and no excitation at all (No excitation). The x-axes are centered around the excitation frequency $f_{{{\text{ex}}}}$ which equals the resonance frequency of the respective resonance mode. The results are shown for (**e**) the 1st bending mode (RM1) and (**f**) the first higher resonance mode (RM2).

In the following, all measurements are performed at *B* = 0. An important parameter for the maximum voltage sensitivity $S_{{\text{V}}}$ and the optimum limit of detection *LOD*, is the excitation voltage amplitude $\hat{u}_{{{\text{ex}}}}$. To find the optimum $\hat{u}_{{{\text{ex}}}}$, the sensor is operated in RM2 at the determined operating point of $f_{{{\text{ex}}}} = 47.96{\text{ kHz}}$ and *B* = 0 for increasing $\hat{u}_{{{\text{ex}}}}$. A sinusoidal magnetic test signal $B_{{{\text{ac}}}} (t)$ with an amplitude of $\hat{B}_{{{\text{ac}}}} = {\text{100 nT}}$ and a frequency of $f_{{{\text{ac}}}} = {\text{10 Hz}}$ is applied. The sensor’s output signal is demodulated to obtain the voltage amplitude $\hat{u}$, required to calculate the voltage sensitivity $S_{{\text{V}}}$. Noise measurements are performed at each $\hat{u}_{{{\text{ex}}}}$ to obtain the demodulated voltage noise density *U*, necessary to estimate the *LOD* with Eq. (6). The results are plotted in Fig. 4a for $S_{{\text{V}}}$ and *U,* and in Fig. 4b the *LOD* as a function of $\hat{u}_{{{\text{ex}}}}$. It can be seen that, in accordance with Eq. (5), $S_{{\text{V}}}$ increases linearly with $\hat{u}_{{{\text{ex}}}}$, consequently $S_{{{\text{am}}}}$ is approximately constant with $\hat{u}_{{{\text{ex}}}}$. In contrast to the voltage sensitivity, the voltage noise density *U* increases more than linearly with $\hat{u}_{{{\text{ex}}}}$. We obtain a minimum *LOD* (at 10 Hz and $\hat{u}_{{{\text{ex}}}} = 650{\text{ mV}}$) of about $LOD = 450{\text{ pT/}}\sqrt {{\text{Hz}}}$. This signal-and-noise behavior has been shown previously on similar sensors without exchange bias^25^, where the minimum LOD was slightly worse, but at a similar excitation amplitude. It was argued that magnetic noise is induced by inverse magnetostriction during the cantilever oscillation and therefore increases with the excitation amplitude. The magnetic noise has been linked directly to magnetic domain activity in other magnetoelectric sensor devices, which might also apply here^26^.

Delta-E signal-and-noise measurements are performed at the optimum operating conditions of $\hat{u}_{{{\text{ex}}}} = 650{\text{ mV}}$ and *B* = 0 with frequencies of the magnetic test signal between $f_{{{\text{ac}}}} = 1 - 300{\text{ Hz}}$ and an amplitude of $\hat{B}_{{{\text{ac}}}} = 100{\text{ nT}}$. The measured output amplitude $\hat{u}$ and the corresponding voltage sensitivity $S_{{\text{V}}}$ [Eq. (5)] as functions of the signal frequency $f_{{{\text{ac}}}}$ are shown in Fig. 4c. As observed in sensors without exchange bias^25^ the output amplitude decreases with $f_{{{\text{ac}}}}$ due to the low pass characteristic of the mechanical resonator. At about 100 Hz, an additional maximum is present in the data that correlates with the appearance of the third resonance mode RM3 seen in the admittance measurements. With the voltage sensitivity $S_{{\text{V}}}$, the detection limit (LOD) is calculated as a function of $f_{{{\text{ac}}}}$ [Eq. (6)] and plotted in Fig. 4d. We obtain an $LOD_{2} \le 450{\text{ pT/}}\sqrt {{\text{Hz}}}$ in the range of 10 – 50 Hz, with a minimum of about $350{\text{ pT/}}\sqrt {{\text{Hz}}}$ at 25 Hz. These detection limits are in the range of $100 - 500{\text{ pT/}}\sqrt {{\text{Hz}}}$ as reported for externally biased sensors of a comparable geometry^22,23,25^.

### Dual-mode operation

In this section, the sensor is operated with a scheme we refer to as dual-mode operation. In dual-mode operation, we localize the sensor using the direct detection scheme and simultaneously measure small amplitude and low-frequency magnetic fields via the delta-E effect. To distinguish the signals picked up by the respective operation scheme from each other, two different resonance modes are used. For the direct detection, the small bandwidth 1^st^ bending mode (RM1) is excited. For the detection of the small-amplitude signal via the delta-E effect, a higher-order resonance mode is used to benefit from the larger bandwidth. In this example, we use the RM2 mode for the delta-E measurement, which was thoroughly analyzed in the previous section.

In the following, the influence of the parallel operation on the noise floor is analyzed. Measurements of the sensor are performed in four different ways as shown in Fig. 4e and f. First, it is operated in direct detection in the first bending mode (Only ME). Secondly, it is operated only in the delta-E read-out scheme in RM2 with a 10 Hz test signal (Only $\Delta E$). Thirdly, both modes are operated simultaneously (Dual-Mode). For the delta-E excitation voltage, we use the optimum value of $\hat{u}_{{{\text{ex}}}} = 650{\text{ mV}}$ and for the magnetic excitation of the first bending mode a field amplitude of $\hat{B}_{{{\text{ac}}}} \approx 5 \text{ } \upmu {\text{T}}$. Additional to the three measurements the noise floor was quantified without any active excitation (No excitation). The measurements were operated outside of the magnetic shielding in a similar setup as used for the localization in the next section.

As expected, a carrier peak is visible at the excitation frequency of RM1 (Fig. 4e) for only ME and dual-mode operation. Correspondingly, a carrier peak is visible at RM2 (Fig. 4f) for only delta-E and dual-mode operation. In RM2 sidebands are visible at $\pm 10{\text{ Hz}}$ around the carrier. They result from the modulation of the sensor current by the 10 Hz magnetic test signal via the delta-E effect [Eq. (2)]. In RM1 the smaller bandwidth results in a larger attenuation of the low-frequency test signal compared to the higher frequency RM2 mode. Additionally, the carrier amplitude is more than two orders of magnitude smaller than in RM2 and hence also the sidebands. Consequently, no sidebands are visible in RM1, although the delta-E modulation does occur. The suppression of sidebands below the noise level is advantageous for simultaneous measurement and localization in dual-mode operation. It simplifies the signal processing by separating the source signal from the localization ME-signal in the frequency regime. The main requirement for the localization is a clear separation of the ME-signal from the noise floor. Hence, the ME-signal can be tuned within a large amplitude range to provide a measurable signal with still sufficient sideband suppression.

### Dual-mode measurements with localization

**Figure 5:**
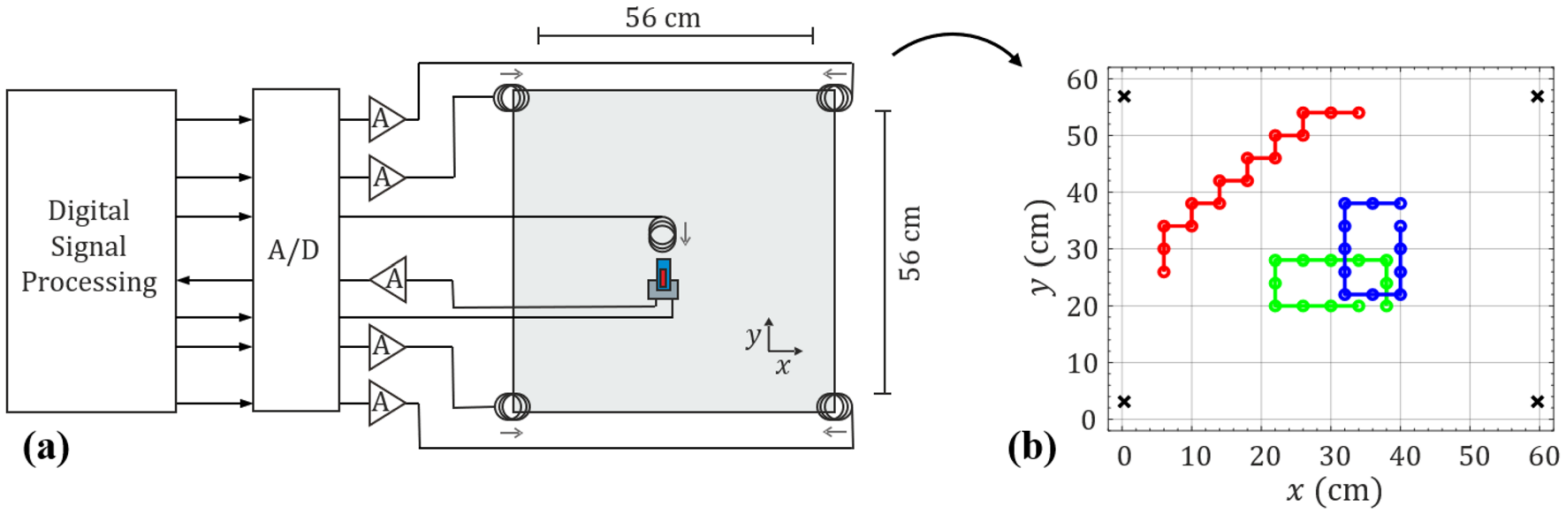
Dual-mode and localization test setup. (**a**) Measurement setup for the dual-mode operation of the exchange bias ME sensors. Four coils are positioned around the measurement area for the localization of the ME sensor and one additional coil is generating a magnetic test signal. The orientations of the coils are denoted by the grey arrows. The source coil is always oriented towards the sensor and shown here for the example of the sensor being oriented along the y-axis. (**b**) Sensor positions used for testing the localization of the ME sensor. Three different sets of positions (green, blue, and red circles) have been tested. The black crosses mark the positions of the transmitting coils. In total, 39 different positions were tested. With two different orientations each this results in a total of 78 different position-orientation pairs.

The measurements were performed outside of the magnetically shielded setup using the sensor analyzed before and four coils transmitting orthogonal signals for the localization of the sensor. Two of the coils were oriented in the positive x-direction and the other two coils in the negative x-direction. All coils were calibrated, and the coil signals were amplified after D/A conversion. An additional coil generates a low-frequency magnetic test signal with frequency components similar to those of a human heart signal. It is to be measured in the delta-E operation mode in RM2. The coil is oriented towards the sensor with a fixed distance and an amplitude of the r-wave of approximately 250 nT at the sensor’s position. A sketch of the measurement setup is shown in Fig. 5a. The position and orientation of the sensor is estimated by solving an inverse problem using a minimum least squares algorithm^43^. A forward model was designed considering the positions and orientations of the coils as well as various position-orientation pairs the sensor could occupy. The coils were approximated by a magnetic point dipole model^44^. Details on the localization algorithm and the calculation of the forward model can be found in the “Materials and methods” section.

**Figure 6:**
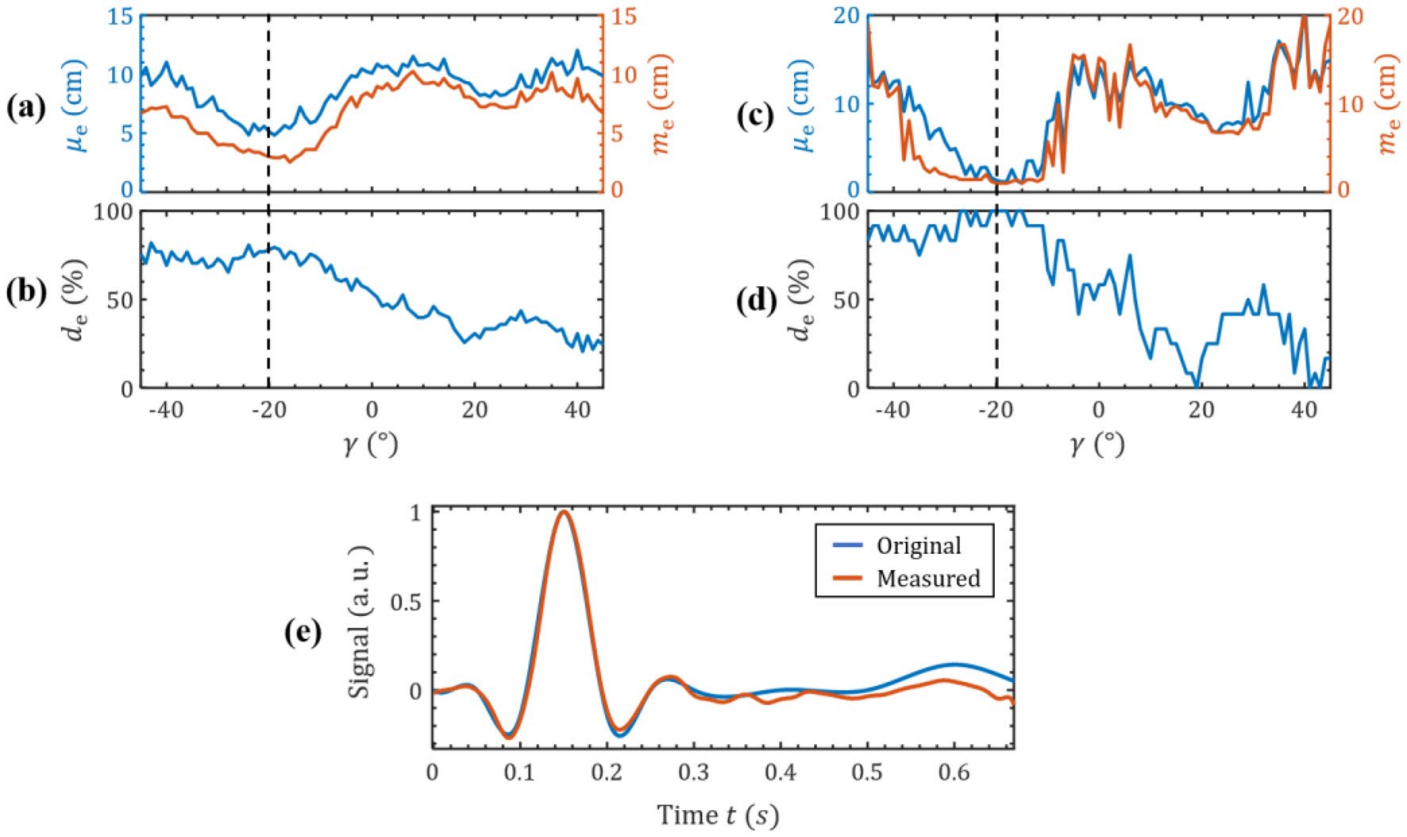
Localization and dual-mode measurement. Average localization error as a function of the tilt $\gamma$ of the sensor’s sensitivity axis for the localization for all of the tested position-orientation-pairs shown in Fig. 5b. In (**a**) the mean $\mu_{{\text{e}}}$ and median $m_{{\text{e}}}$ Euclidean distance between real and estimated sensor positions and in (**b**) the percentage of correct estimated sensor orientations are shown. (**c**) Average localization error as a function of $\gamma$ for the localization of the blue set of positions in Fig. 5b, (**c**) The mean $\mu_{{\text{e}}}$ and median $m_{{\text{e}}}$ Euclidean distance between real and estimated sensor positions and in (**d**) the percentage of correct estimated sensor orientations is shown. (**e**) Demodulated, detrended, lowpass-filtered and normalized magnetic test signal measured by the ME sensor in the 2^nd^ bending mode and original normalized transmitted signal. Due to the larger bandwidth of the sensor in resonance mode RM2 the signal can be reconstructed well.

In this study we use a $56 \times 56\;{\text{cm}}^{2}$ test area with a grid size of 1 cm used for the minimum least squares algorithm. The long axis of the sensor is always oriented along one of the unit vectors $e_{{\text{x}}}$ and $e_{{\text{y}}}$ of the Cartesian coordinate system. Three different sets of positions were tested (Fig. 5b), each set for both orientations. This results in a total of 78 different position-orientation pairs measured. These position-orientation-pairs were localized using different tilts $\gamma$ of the sensor’s sensitivity axis relative to the long axis of the sensor in the forward problem. The tilts were varied between -45° and 45° in 1° steps. The mean magnitude position estimation error $\mu_{{\text{e}}}$ as well as the median magnitude position estimation error $m_{{\text{e}}}$ and the percentage of correct estimated sensor orientations $d_{{\text{e}}}$ for all the tested position-orientation-pairs are shown in Fig. 6a,b.

A minimum of the localization error results for a tilt of $\gamma \approx 20^\circ \pm 10^\circ$ of the sensor’s sensitivity axis. One reason for this broad minimum is the robustness of the localization algorithm against smaller orientation mismatches. The results show a minimum mean localization error of about $\mu_{{\text{e}}} = 4.82\;{\text{cm}}$ and a minimum median error of about $m_{{\text{e}}} = 2.532{\text{ cm}}$ for the positions shown in Fig. 5b. The large difference between $\mu_{{\text{e}}}$ and $m_{{\text{e}}}$ indicates outliers in the localization results. This error depends on the positions measured. Considering, for instance, only the blue set of sensor locations (Fig. 5b) with orientations in $e_{{\text{y}}}$ led to better results as shown in Fig. 6c-d. The localization error is $\mu_{{\text{e}}} = 0.991\;{\text{cm}}$ and $m_{{\text{e}}} = 1{\text{ cm}}$. The results depicted in Fig. 6a–d are comparable with the results of other magnetic localization approaches, where no signal was measured simultaneously. For instance, in^45^ an average error of 5.3 cm could be achieved by spanning a $3 \times 3{\text{ m}}^{2}$ grid with three transmitter coils. In^46^ a 1D coil and a 3D sensor were used for localization. The grid size was $8 \times 7{\text{ cm}}^{2}$ for the 2D localization case and the average localization error was about 2 mm.

For the proof of concept, only simple models of the coils and the sensor were used here. With improved models, based on the Biot-Savart-Law for the coils and a more accurate model for the sensor that considers its geometry we anticipate a significant reduction of the localization error^10^. Further improvement could be achieved by e.g. increasing the number of transmitting coils to reduce ambiguities. The demodulated signal of RM2 for an example dual-mode measurement is depicted in Fig. 6e. Due to the higher bandwidth of RM2 the magnetic test signal can be almost entirely reconstructed. After demodulation, the signal is lowpass-filtered with a cut-off frequency of 30 Hz for the removal of noise and linearly detrended.

## Summary and conclusion

We presented a fully integrable low-field delta-E effect sensor with MnIr-based exchange biased multilayer. It was thoroughly analyzed, and its potential was demonstrated with a dual-mode operation technique for simultaneous measurement and localization. A magnetic domain model was extended to analyze the magnetic properties of an example sensor. The model matches the magnetization curves well over the full angular range of the applied magnetic field. It confirms the implementation of an internal bias field at an angle $\varphi_{{{\text{ex}}}} = 46^\circ$ and a magnitude $B_{{{\text{ex}}}} \approx 0.8{\text{ mT}}$.

For the localization of the sensor, the first bending mode was analyzed in the direct detection mode. At zero bias field, a detection limit of $LOD_{{{\text{ME}}}} = 150{\text{ pT/}}\sqrt {{\text{Hz}}}$ is found in mechanical resonance at $f_{{{\text{r}},1}} = 7.728{\text{ kHz}}$ with brass cylinder, and *LOD*_ME_ = 30 pT/Hz without. This is higher than measured with externally biased, cm-sized sensors^17,47^ optimized for the direct detection, but significantly improved compared to mm-sized sensors^36^. A detection limit in the low pT-regime is not required here, because the direct detection mode is only used for the sensor localization.

Two higher resonance modes (RM2 and RM3) are found around 48 kHz and analyzed for the delta-E operation. Compelling evidence from the models and measurements suggests that RM2 is the first torsional mode and RM3 the second bending mode. The measurements of RM2 match with the magnetic delta-E model. The quality factor at zero bias field of the RM3 mode $Q_{3} = 1100$ is around typical values of $Q \approx 1000$^22,23,25^ for bending modes, whereas $Q_{2} = 1400$ of RM2 is notably higher. At the optimum excitation voltage and *B* = 0, detection limits of $LOD_{2} \le 450{\text{ pT/}}\sqrt {{\text{Hz}}}$ (RM2) are measured in the range between 10 and 50 Hz, with a minimum of about $350{\text{ pT/}}\sqrt {{\text{Hz}}}$ at 25 Hz. Overall, we demonstrated that delta-E sensor characteristics in the same range of externally biased devices^22,23,25^ are possible with exchange biased multilayers on small sensors. Hence, problems detrimental to the application which arise from using coils are avoided.

Operating the sensor in dual-mode was shown to have no measurable impact on the detectivity of the signal detection in delta-E effect mode (RM2), for the excitation amplitudes used. Vice-versa, additional magnetic noise from the delta-E operation does slightly increases the noise floor in the lower frequency regime, which is used for the direct detection (RM1). Because the power of the localization signal can always be adjusted to provide a sufficient signal-to-noise ratio the additional noise is not detrimental for the localization. It was also shown that sidebands that would occur around RM1 from the delta-E modulation are naturally suppressed by the small bandwidth and the small carrier amplitude of the lower frequency RM1 mode. This is key for simultaneous measurement and localization in dual-mode operation. It significantly simplifies the signal processing by separating the source signal from the localization signal in the frequency domain. We demonstrated the feasibility of this concept and its application for the simultaneous sensor localization and measurement of a low-frequency magnetic signal. Mean localization errors between approximately 1–5 cm were obtained and mean median errors between 1 and 2.5 cm. These promising results were achieved despite the simple models used for the coils and the sensor in the forward problem. More accurate models are anticipated to reduce the localization error further^10^. The current results are comparable with those of other magnetic localization approaches, where no signal was measured simultaneously^45,46^.

In conclusion, we presented and analyzed the first exchange biased delta-E effect sensor. With the internal bias, severe problems are avoided that accompany the usage of external coils. Dense, large number sensor arrays are now potentially feasible and a significant step towards real applications is made. Additionally, the concept of the dual-mode operation technique was demonstrated. The combination of both enables the detection of extremely small, low-frequency magnetic fields while localizing the sensor. The simultaneous localization might also bear the potential to reduce errors from source movements during the measurement and will be investigated with this regard in the future.

## Materials and methods

### Sensor fabrication

The senor is fabricated starting from a 700 µm oxidized double side polished silicon wafer, covered by 50 µm polysilicon and another 650 nm oxide layer. All functional layers are deposited by magnetron sputtering from a von Ardenne CS730 S and structured by optical lithography and a combination of wet and dry etching techniques. The magnetic layer is deposited first and made of a sequence of $20 \times ({\text{Ta / Cu / Mn}}_{70} {\text{Ir}}_{30} /{\text{Fe}}_{70.2} {\text{Co}}_{7.8} {\text{Si}}_{12} {\text{B}}_{10} )$ with individual layer thicknesses of Ta 5 nm, Cu 3 nm, MnIr 8 nm and FeCoSiB 200 nm. For a detailed structural characterization see^48^. Ion beam etching is used to structure the magnetic layer. A bottom electrode of Ta 40 nm Pt 150 nm is deposited over the whole cantilever, also serving as seed layer for the following piezoelectric layer (AlN, 2 µm), which is deposited without additional heating by a pulsed DC generator as published in^31^. The AlN is structured by 80 °C H_3_PO_4_ (85%). After the lift off formation of the 150 nm thick Ta/Pt top electrodes (E1: 0.2 mm × 1 mm, E2: 0.38 mm × 0.2 mm), the silicon carrier wafer is etched with TMAH (25%) at 80 °C, leaving a silicon frame off 7.5 mm × 11 mm for handling. The cantilever (width 1 mm, length 3 mm) is released by deep reactive ion etching using a modified Bosch-Process in a Sentech SI500 ICP-RIE. After separating the MEMS Chips by dicing saw, the sensors are annealed in an oil bath at 250 °C for 40 min with an applied field of 55° with respect to the length axis of the cantilever.

### Magnetization measurements

To analyze the magnetic properties of the device, measurements were performed with a BH-loop tracer at 16 Hz and angles $\varphi_{{\text{H}}}$ from 0° to 345° in steps of 15° relative to the sensor’s long axis. The applied magnetic field was swept from -10 mT to 10 mT.

### Signal and noise measurements

All electrical measurements are performed with a high-resolution A/D and D/A converter *Fireface UFX* + (*RME*, Germany) in a magnetically shielded setup^49^ if not stated differently. The charge amplifier used has a feedback capacitance ${\text{C}}_{{\text{f}}} = {33}\;{\text{pF}}$ and a feedback resistance $R_{{\text{f}}} = 5{\text{ G}}\Omega$. The signal and noise measurements in Figs. 2 and 4 are performed six times for 4 min and then averaged in the frequency domain over segments of 5 s to obtain a smooth noise floor. The measurements in Fig. 4e and f and Fig. 6 are performed outside of the magnetically shielded setup. Each signal was measured over 1 min and averaged in the frequency domain using segments of 5 s to obtain a smooth noise floor and to better show the influence of the dual-mode operation scheme.

### Numerical eigenfrequency study

For the calculation of the first three eigenfrequencies of the cantilever we use an eigenfrequency study in *COMSOL Multiphysics* *5.4*. The simplified model geometry is a rectangular beam with in-plane dimensions of $3{\text{ mm}} \times {\text{1 mm}}$ and fixed boundary conditions at the left end. The model cantilever consists of only two layers: one for the magnetic (FeCoSiB) layer and one substrate layer (Substrate) that includes all other layers via effective material properties. The material parameters used are given in the following table (Table 2). For the Young’s modulus of the magnetic layer we consider the delta-E effect, using a value smaller than its saturation value of $E_{{\text{m}}} \approx 150{\text{ GPa}}$. The values used are consistent with material parameters used previously^38^. Slight differences can occur from stress, curvature and process related deviations from the targeted geometry^15^.

**Table 2:** Model parameters. Layer thicknesses *t*, Young’s modulus *E*, Poisson’s ratio $\nu$ and density $\rho$ used in the finite element simulation.

	*t* [µm]	*E* [GPa]	$\nu$	$\rho \text{ }$[kg/m^3^]
Substrate	52	160	0.22	2340
FeCoSiB	4	140	0.35	7900

### Damped harmonic oscillator fit

The resonance amplitude $\hat{X} = \left| {X(\omega )} \right|$ of the oscillator model is fitted to the data and obtained from the frequency response

7 $$X(\omega ) = \frac{{A_{{\text{m}}} }}{{c_{{\text{m}}} i\omega + \omega_{0}^{2} - \omega^{2} }},$$

with the angular frequency $\omega = 2\pi f$, the angular eigenfrequency $\omega_{0}$, and the complex number $i = \sqrt { - 1}$. The oscillation amplitude $A = A_{{\text{m}}} m$ and the damping constant $c = c_{{\text{m}}} m$ are both normalized to the effective mass *m*. The parameters $\omega_{0}$, $A_{{\text{m}}}$ and $c_{{\text{m}}}$ are obtained from the fit. The effective mass *m* is calculated using $\omega_{0}$ and the effective spring constant *k*

8 $$m = \frac{{\omega_{0}^{2} }}{k}{\text{ with }}k = \frac{{3(EI)_{{{\text{eff}}}} }}{{L^{3} }}.$$

The term $(EI)_{{{\text{eff}}}} : = E_{1} I_{1} + E_{2} I_{2}$ is the effective bending stiffness for the same simplified 2-layer cantilever geometry considered in the finite element model (“Numerical eigenfrequency study”). In $(EI)_{{{\text{eff}}}}$ the Young’s moduli of the substrate and of the FeCoSiB film are given by $E_{1}$, $E_{2}$ and the corresponding second moments of area by $I_{1}$ and $I_{2}$. The quality factor $Q = 1/(2\xi )$ is then calculated from the damping ratio

9 $$\xi = \frac{c}{{2\sqrt {mk} }} = \frac{c}{{2\sqrt {\omega_{0}^{2} } }}.$$

### Exchange bias magnetic domain model

In this section we extend a numerical domain model^30^ by including an exchange bias and arbitrary external magnetic field direction. In the model a pair of simplified 180° domains is considered, consisting of two magnetic moments and a movable domain wall. It represents an average configuration of all domains in the sample. The original total enthalpy density $u$ of the system is extended by an exchange anisotropy energy density $u_{{{\text{ex}}}}$ and a more general Zeeman energy density term $u_{{\text{Z}}}$. The total enthalpy density $u$ is

10 $$u = u_{{\text{K}}} + u_{{\text{Z}}} + u_{{{\text{ex}}}} + u_{{{\text{me}}}} + u_{{\text{w}}} .$$

The expressions used for the effective uniaxial anisotropy $u_{{\text{K}}}$, the magnetoelastic anisotropy energy density $u_{{{\text{me}}}}$ and the phenomenological domain wall term $u_{{\text{w}}}$ can be found in^30^. For $u_{{\text{Z}}}$ we use the magnetic vacuum permeability $\mu_{0}$, saturation magnetization $M_{{\text{s}}}$, magnitude *H* and angle $\varphi_{{\text{H}}}$ of the external magnetic field vector $\overline{H}$. It is

11 $$u_{{\text{Z}}} = \mu_{0} M_{{\text{s}}} H \cdot \left[ {\nu_{2} \cos (\theta + \varphi_{2} - \varphi_{{\text{H}}} ) - \nu_{1} \cos (\theta - \varphi_{1} - \varphi_{{\text{H}}} )} \right]$$

with the angles $\varphi_{1}$ and $\varphi_{2}$ of $m_{1}$ and $m_{2}$ relative to the easy axis with angle $\theta$. All angles except of $\varphi_{1}$ and $\varphi_{2}$ are defined relative to the long axis of the cantilever (x-axis). The contribution of each domain is weighted by its respective volume fraction $\nu_{1}$ and $\nu_{2}$. The exchange anisotropy energy density is defined via the magnitude $H_{{{\text{ex}}}}$ and the angle $\varphi_{{{\text{ex}}}}$ of the exchange bias field vector to the x-axis and results to

12 $$u_{{{\text{ex}}}} = \mu_{0} M_{{\text{s}}} H_{{{\text{ex}}}} \cdot \left[ {\nu_{2} \cos (\theta + \varphi_{2} - \varphi_{{{\text{ex}}}} ) - \nu_{1} \cos (\theta - \varphi_{1} - \varphi_{{{\text{ex}}}} )} \right] \, {.}$$

Following the method in^30^ we obtain the normalized wall displacement equation

13 $$\frac{{x_{{\text{w}}} }}{d} = \frac{1}{{2Ad^{2} w\sin^{2} \theta }} \cdot \left[ {\mu_{0} M_{{\text{s}}} H_{{{\text{ex}}}} \cdot C + \mu_{0} M_{{\text{s}}} H \cdot D - \frac{3}{2}\lambda_{{\text{s}}} \sigma \cdot E - K \cdot F} \right]{ ,}$$

with

14 $$\begin{gathered} C: = \cos (\theta - \varphi_{1} - \varphi_{{{\text{ex}}}} ) + \cos (\theta + \varphi_{2} - \varphi_{{{\text{ex}}}} ) \, , \hfill \\ D: = \cos (\theta - \varphi_{1} - \varphi_{{\text{H}}} ) + \cos (\theta + \varphi_{2} - \varphi_{{\text{H}}} ) \, , \hfill \\ E: = \sin^{2} (\theta - \varphi_{1} ) - \sin^{2} (\theta + \varphi_{2} ) \, , \hfill \\ F: = \sin^{2} (\varphi_{1} ) - \sin^{2} (\varphi_{2} ) \, . \hfill \\ \end{gathered}$$

The initial domain width is denoted as *d*. Having the wall displacement Eqs. (13), (10) can be minimized numerically to obtain the residual unknowns $\varphi_{1}$ and $\varphi_{2}$. The projection of the reduced magnetization *m* on the axis of the external magnetic field is then obtained from

15 $$m = \nu_{1} \cos (\theta - \varphi_{1} - \varphi_{{\text{H}}} ) - \nu_{2} \cos (\theta + \varphi_{2} - \varphi_{{\text{H}}} ) \, .$$

With the Young’s modulus $E_{{\text{m}}}$ at fixed magnetization, the effective magnetization dependent Young’s modulus can be described by^20^

16 $$E = \left[ {\frac{\partial e}{{\partial \sigma }} + \frac{\partial \lambda }{{\partial \sigma }}} \right]^{ - 1} : = \left[ {\frac{1}{{E_{{\text{m}}} }} + \frac{1}{\Delta E}} \right]^{ - 1} { ,}$$

For the magnetostrictive part we obtain

17 $$\begin{gathered} \frac{1}{\Delta E} = \nu_{1} \frac{{9\lambda_{{\text{s}}}^{2} \sin^{2} (2\left[ {\theta - \varphi_{1} } \right])}}{{4u_{{\varphi \varphi_{1} }} }} \hfill \\ - \nu_{2} \frac{{9\lambda_{{\text{s}}}^{2} \sin^{2} (2\left[ {\theta + \varphi_{2} } \right])}}{{4u_{{\varphi \varphi_{2} }} }} \, , \hfill \\ \end{gathered}$$

with the second order derivatives $u_{{\varphi \varphi_{1,2} }}$

18 $$\begin{aligned} u_{{\varphi \varphi_{1} }} &= \nu_{1} \left[ {3\lambda_{{\text{s}}} \sigma \cos \left( {2\left[ {\theta - \varphi_{1} } \right]} \right)} \right. + 2K\cos \left( {2\varphi_{1} } \right) \hfill \\ &\quad + \mu_{0} M_{{\text{s}}} H_{{{\text{ex}}}} \cos \left( {\theta - \varphi_{1} - \varphi_{{{\text{ex}}}} } \right) \hfill \\ &\quad + \left. {\mu_{0} M_{{\text{s}}} H\cos \left( {\theta - \varphi_{1} - \varphi_{{\text{H}}} } \right)} \right] , \end{aligned}$$

and

19 $$\begin{aligned} u_{{\varphi \varphi_{2} }} &= - \nu_{2} \left[ {3\lambda_{{\text{s}}} \sigma \cos \left( {2\left[ {\theta + \varphi_{2} } \right]} \right)} \right. + 2K\cos \left( {2\varphi_{2} } \right) \hfill \\ &\quad + \mu_{0} M_{{\text{s}}} H_{{{\text{ex}}}} \cos \left( {\theta + \varphi_{2} - \varphi_{{{\text{ex}}}} } \right) \hfill \\ &\quad + \left. {\mu_{0} M_{{\text{s}}} H\cos \left( {\theta + \varphi_{2} - \varphi_{{\text{H}}} } \right)} \right] . \end{aligned}$$

For the simulations a saturation magnetostriction of $\lambda_{{\text{s}}} = 35{\text{ ppm}}$ is used and a saturation flux density of $\mu_{0} M_{{\text{s}}} = 1.5{\text{ T}}$^50^. A wall stiffness parameter of $w = {5} \cdot {10}^{8} {\text{ J/m}}^{4}$ is found from the fit and an initial domain wall width of $d = 50 \text{ }\upmu{\text{m}}$ is used.

### Analytical eigenfrequency calculation

The Eigenfrequency $f_{{\text{r,n}}}$ of the $n^{th}$ bending mode of a Euler–Bernoulli beam is given by^22^

20 $$f_{{\text{r,n}}} = \frac{{\lambda_{n}^{2} }}{{2\pi L^{2} }}\sqrt {\frac{{\sum\nolimits_{k} {E_{k} I_{k} } }}{{\sum\nolimits_{k} {\rho_{k} A_{k} } }}} ,$$

with the mode factor $\lambda_{i}$, the cantilever length *L* the mass density $\rho_{k}$, cross section area $A_{k}$ the Young’s modulus $E_{k}$ and the second moment of area $I_{k}$ all of the $k^{th}$ layer. For the second bending mode it is $\lambda_{2} = 4.694$. The material and geometry parameters are identical to those used in the eigenfrequency study in Table 2. The Young’s modulus of the substrate was slightly adjusted to 162.7 GPa to match the measured resonance frequency of RM3. The Young’s modulus of the magnetic layer as a function of the applied flux density is obtained from the domain model [Eqs. (16)–(19)] using a saturation Young’s modulus of $E_{{\text{m}}} = 150{\text{ GPa}}$.

### Localization algorithm

For the localization of the sensor, coils positioned at the edge of the localization area transmit artificial signals. The coils used for localization consist of approximately 350 windings and have a radius of about 1.25 cm. The maximum rms value of the coil excitation current was about 164 mA. Position and orientation of the sensor can be inferred from the signals received at the sensor by solving an inverse problem. The magnetic flux density of the coil at the sensor’s position is approximated by a magnetic dipole field^44^

21 $$\overline{B}_{i} (t) = \frac{{\mu_{0} }}{4\pi } \cdot \frac{{3\left[ {\overline{r}_{{\text{s}}} - \overline{r}_{{{\text{c}}_{i} }} } \right]\left[ {\left. {\overline{m}_{i} (t)} \right|\left( {\overline{r}_{{\text{s}}} - \overline{r}_{{{\text{c}}_{i} }} } \right)} \right] - \overline{m}_{i} (t)\left\| {\overline{r}_{{\text{s}}} - \overline{r}_{{{\text{c}}_{i} }} } \right\|_{2}^{2} }}{{\left\| {\overline{r}_{{\text{s}}} - \overline{r}_{{{\text{c}}_{i} }} } \right\|_{2}^{5} }}$$

with the permeability of vacuum $\mu_{0}$, the position $\overline{r}_{{\text{s}}}$ of the sensor, the position $\overline{r}_{{{\text{c}}_{i} }}$ of the coil *i* and the magnetic dipole moment $\overline{m}_{i} (t)$. The operator $\left\langle {{\overline{x}}} \mathrel{\left | {\vphantom {{\overline{x}} {\overline{y}}}} \right. \kern-\nulldelimiterspace} {{\overline{y}}} \right\rangle$ denotes the scalar product of the vectors $\overline{x}$ and $\overline{y}$. Equation (21) is a good approximation if the distance between the transmitting coil and the sensor is large enough^44^. The sensitivity of the sensor is anisotropic and thus the measured signal

22 $$u(t) = h_{{\text{s}}} (t) * \left\langle {{\overline{d}_{{\text{s}}} }} \mathrel{\left | {\vphantom {{\overline{d}_{{\text{s}}} } {\sum\nolimits_{i = 1}^{N} {\overline{B}_{i} (t)} }}} \right. \kern-\nulldelimiterspace} {{\sum\nolimits_{i = 1}^{N} {\overline{B}_{i} (t)} }} \right\rangle$$

is the scalar product of the directivity $\overline{d}_{{\text{s}}}$ of the sensor with the superposition of all *N* coil signals and a convolution with the impulse response $h_{{\text{s}}} (t)$ of the sensor system including the charge amplifier. This equation is valid at least for the considered frequency range around the first bending mode. To determine the signal components of each coil the transmitted signals must be orthogonal^51^. This is achieved here with a Time Division Multiple Access (TDMA) approach^52^. Moreover, the frequency of the signals should be as close as possible to the resonance frequency of the resonance mode used to benefit from the sensor’s high sensitivity in resonance. Thus, the coil *i* transmits the sinusoidal signal

23 $$s_{i} (t) = \cos \left( {2\pi f_{{\text{r,1}}} \left[ {t - \tau_{i} } \right]} \right) \cdot w\left[ {t - \tau_{i} } \right]{\text{ with }}\tau_{i} = \left[ {i - 1} \right] \cdot \left[ {T_{{{\text{coil}}}} + T_{{\text{p}}} } \right]$$

with the resonance frequency $f_{{\text{r,1}}}$ of the sensor in the first bending mode, the transmitting time $T_{{{\text{coil}}}}$ of the signal, and the pausing time $T_{{\text{p}}}$ between two signals. The quantity *w(t)* corresponds to a Hann window function of length $T_{{{\text{coil}}}}$. The localization area is divided into *M* different positions and orientations the sensor could occupy $\overline{P} = \left[ {\overline{p}^{1} ,...,\overline{p}^{j} ,...,\overline{p}^{M} } \right]$ and a forward solution generating a lead field matrix $\overline{A}$ for these possible states is calculated. The lead field matrix consists of the signal component that each coil would contribute if the sensor occupied the possible position and orientation. The inverse solution is determined by solving a minimum least squares problem

24 $$\mathop {\min }\limits_{j} \sum\limits_{i = 1}^{N} {\left( {\frac{{\left| {\overline{A}(i,j)} \right|}}{{\left| {\overline{A}(i_{{{\text{max}}}},j )} \right|}} - \frac{{\left| {\overline{\hat{s}}(i)} \right|}}{{\left| {\overline{\hat{s}}(i_{{{\text{max}}}} )} \right|}}} \right)^{2} }$$

comparable with the one presented in^43^. The lead field matrix entries of each possible position-orientation-combination *j* are compared with the estimated signal components $\overline{\hat{s}}$ of the coils measured by the sensor. Due to the sinusoidal excitation and the comparison of ratios the impulse response of the sensor denoted in Eq. (21) can be neglected. The signal components are extracted by applying a matched filter^53^. The lead field matrix entries as well as the extracted signal components are normalized by the entry $i_{{{\text{max}}}}$. The entry $i_{{{\text{max}}}}$ corresponds to the maximal absolute signal component. The position and orientation leading to the best fitting entry $j_{{{\text{max}}}}$ are most likely the position and orientation of the sensor $\overline{\hat{p}}_{{\text{s}}} = \overline{p}^{{j,{\text{min}}}}$.
